# Elevated production of the aromatic fragrance molecule, 2‐phenylethanol, using Metschnikowia pulcherrima through both de novo and ex novo conversion in batch and continuous modes

**DOI:** 10.1002/jctb.5597

**Published:** 2018-03-25

**Authors:** Tanakorn Chantasuban, Fabio Santomauro, Deborah Gore‐Lloyd, Sophie Parsons, Daniel Henk, Roderick J Scott, Christopher Chuck

**Affiliations:** ^1^ Department of Chemical Engineering University of Bath UK; ^2^ Department of Biology & Biochemistry University of Bath UK; ^3^ Department of Mechnical Engineering University of Bath UK

**Keywords:** Adsorption, Biochemical engineering, Bioprocesses, Engineering, Green Engineering/Products, Industrial biotechnology

## Abstract

**BACKGROUND:**

2‐phenylethanol (2PE) is a fragrance molecule predominantly used in perfumes and the food industry. It can be made from petrochemicals inexpensively, however, this is unsuitable for most food applications. Currently, the main method of production for the bio‐derived compound is to extract the trace amounts found in rose petals, which is extremely costly. Potentially fermentation could provide an inexpensive, naturally sourced, alternative.

**RESULTS:**

In this investigation, 2PE was produced from the yeast Metschnikowia pulcherrima, optimised in flasks before scaling to 2 L batch and continuous operation. 2PE can be produced in high titres under de novo process conditions with up to 1500 mg L^−1^ achieved in a 2 L stirred bioreactor. This is the highest reported de novo titre to date, and achieved through high sugar loadings coupled with low nitrogen conditions. The process successfully ran in continuous mode also, with a concentration of 650 mg L^−1^ of 2PE being maintained. The 2PE production was further increased by the ex novo conversion of phenylalanine and semi‐continuous solid phase extraction from the supernatant. Under optimal conditions 14 000 mg L^−1^ of 2PE was produced.

**CONCLUSIONS:**

The work presented here offers a novel route to naturally sourced 2PE through a scalable fermentation with a robust yeast highly suited to industrial biotechnology. © 2018 The Authors. *Journal of Chemical Technology & Biotechnology* published by John Wiley & Sons Ltd on behalf of Society of Chemical Industry.

## INTRODUCTION

Fragrance compounds are an essential part of food, perfume and personal care products. Traditionally these valuable compounds can be retrieved by extraction from flowers and natural sources which usually require large amounts of biomass where they occur in low concentrations. The yields from natural extraction therefore are very low, which generally makes these fragrances highly expensive. One of the most widely produced single fragrances globally is 2‐phenylethanol (2PE), which has an approximate global production of 10 000 tonnes a year and is an important ingredient in the food and cosmetic industry due to its rose‐like aroma.^1^ 2PE can be produced through a well‐known Friedel–Crafts acylation utilising benzene and ethylene oxide. However, this route produces a mixture of isomers and off‐flavours in the final products making the 2PE unsuitable for human consumption or use in cosmetics.^2^ Naturally derived 2PE extract can cost upwards of $1000 per kg, as the concentration of 2PE in flowers (e.g. rose) is extremely low, the extraction is expensive and complex.^1^ In addition to the poor economics of agrobiological production, which make it unsuitable for meeting the large market demand, naturally derived 2PE extract is not nessessarily environmentally preferable to other technology routes. This relates to the scales required for low extract yield, and agricultural greenhouse gas (GHG) emissions and ecosystems impacts associated with fertiliser and pesticides use.^3^ Despite economic constraints, legal restrictions mean that cosmetic and food manufacturers must use pure 2PE, without isomers and off flavours, derived from natural sources, a policy sustained by an increase in consumer demand for biologically derived additives.^4^

2PE can be produced biochemically in microorganisms via the Ehrlich metabolic pathway using L‐phenylalanine as a precursor. This pathway also includes the biosynthesis of alternative higher alcohols with aromatic amino acids as precursors. Another pathway, the Shikimate pathway, governs the *de novo* synthesis of L‐phenylalanine when L‐phenylalanine in the media is scarce, producing aromatic amino acids from glucose via D‐erythrose‐4‐phosphate.^5^, ^6^ The Shikimate pathway therefore links the metabolism between carbohydrate and amino acid production in several organisms, particularly plants and microorganisms.^5^ Essential aromatic amino acids, such as L‐phenylalanine, L‐tyrosine and L‐tryptophan are created via this pathway, mostly for the synthesis of polypeptides. These amino acids can then be fed into the Ehrlich pathway.^6^, ^7^ Several yeasts, mainly in the genera Kluyveromyces, Zygosaccharomyces, Saccharomycess, Hansenula, Pichia, Candida and Clavispora are reported to be able to produce 2PE.^8^, ^9^, ^10^, ^11^ However, 2PE production from these yeasts is fairly low, usually in the 10–100 mg L^−1^ range, via de novo synthesis (synthesis without additional L‐phenylalanine as a precursor).

Therefore the *de novo* synthesis of 2PE requires the yeast to produce enough phenylalanine, that is then converted in three further steps to 2PE within the cell. As the yields tend to be low, despite only needing a sugar input, it had long been considered that the *de novo* synthesis of 2PE was not economically viable and focus was on the *ex novo* synthesis, using L‐phenylalanine as a substrate instead.^12^ Despite the additional cost of phenylalanine, by adding an excess amount to the culture externally, the yeasts can produce 2PE, simply reducing the feedstock biochemically, which tends to result in far higher yields.

Further improvements in 2PE yield can also be achieved through the *in situ* removal of the resulting 2PE from the fermentation broth. This has the additional benefit of allowing high biomass yields. This was demonstrated by Stark *et al*. to increase the production 10‐fold with extraction with oleic acid.^13^ Solid adsorbents also show promise with the hydrophobic polystyrene resin HZ818 boosting production of 2PE to 6.6 g L^−1^ from *S. cerevisiae*. Similarly, Etschmann and Schrader reported 2PE production of 10.2 g L^−1^ from *K. marxianus* using polypropylene glycol 1200.^14^ The highest titres reported to date have been achieved using the polyester–polyether co‐polymer Hytrel, with 13.7 g L^−1^ obtained from a batch culture.^15^

The *de novo* 2PE biosynthesis could still be commercially exploitable, however, due to the improvement in the depolymerisation of cellulose to produce simple sugars, a raw material cheaper than amino acids,^16^, ^17^, ^18^, ^19^ though higher titres of 2PE must be achieved. In this regard, we recently reported on the yeast *Metschnikowia pulcherrima*, which shows promising characteristics for industrial applications. For example, *M. pulcherrima* can metabolise a range of sugars produced through the depolymerisation of lignocellulose (such as glucose, fructose, xylose, arabinose, cellobiose, lactose). The yeast can be cultured in non‐sterile conditions due to the production of a range of antimicrobial compounds, including 2PE.^20^ *Metschnikowia pulcherrima* has been reported to produce up to 180 mg L^−1^ of 2PE in the hostile conditions present in must during wine fermentation.^8^ As such, this yeast could provide an ideal platform for commercially successful *de novo* production of 2PE.

This study describes the optimisation of the biochemical conditions for the *de novo* 2PE biosynthesis from *M. pulcherrima* and compares this with the *ex novo* production from phenylalanine. On optimisation the process was scaled to 2 L and run in continuous mode with *in situ* extraction of the 2PE to deliver high titres of 2PE.

## MATERIAL AND METHODS

### Materials

All chemicals were supplied by Sigma Aldrich as analytical grade or equivalent unless otherwise stated. *Metschnikowia pulcherrima* (NCYC 373) was obtained from National Collection of Yeast Cultures (Norfolk, UK) and stored on sterile YPD agar plate at 4 °C. The stock culture was re‐plated every two months to ensure culture viability. YMS media used in this report was composed of 30 g L^−1^ yeast extract, 5 g L^−1^ mannitol and 5 g L^−1^ sorbose and used in all *M. pulcherrima* inoculations unless otherwise stated. YPD medium was composed of 10 g L^−1^ yeast extract, 20 g L^−1^ peptone and 20 g L^−1^ glucose and used in agar plate culture in reculturing *M. pulcherrima* stock. Synthetic grape juice (SGJ) media was adapted from the literature,^21^ to replicate grape must in wine fermentation. A further synthetic graph juice media, without fructose, (SGGJ) was also used. The media concentrations are given in the supporting information.

## METHODS

All media and glassware used in *M. pulcherrima* culture were sterilised at 121 °C for 15 min in an autoclave before use. *Metschnikowia pulcherrima* was inoculated from the yeast stock plate in YMS media and incubated at 20 °C and shaking at 150 rpm for 48 h.

### Culture optimisation

For the optimisation of pH, *M. pulcherrima* was inoculated and cultured in 125 mL Erlenmeyer flasks with 25 mL working volume with SGJ with modified pH at 2, 3, 4 and 5 with tartaric and malic acid acting as buffers and internal HPLC standards. The experiments were run in triplicate. The cultures were incubated on a shaker at 180 rpm at 20 °C. Samples were collected daily from the flasks to analyse biomass and a selected sample was analysed by HPLC. For the optimisation of temperature, triplicate cultures were conducted with SGJ media modified to pH 4. The cultures were incubated at 15, 20 and 28 °C. The glucose concentration was optimised through triplicate cultures in SGGJ with no fructose and varying levels of glucose, the N:C ratio was held at 1:100. In the nitrogen/carbon ratio study, *M. pulcherrima* was cultured in SGGJ media with NH_4_(HPO_3_)_2_ as the sole nitrogen source at different concentrations (0.1, 0.2, 0.5 and 1 g N per 100 g carbon). The SGGJ media contained glucose as the sole carbon source (100 g L^−1^). The end products were analysed at the end of fermentation at 7 days.

For the *ex novo* production, *M. pulcherrima* was cultured as described above at pH 4 in SGJ with no nitrogen‐containing compounds, with additional L‐phenylalanine (10 g L^−1^, 20 g L^−1^ and 30 g L^−1^), over 12 days.

The same methodology was used to examine the effect of powdered activated carbon (PAC) on 2PE production, except the *M. pulcherrima* culture contained 0.5 g PAC. The culture was incubated at 20 °C for 7 days. Biomass could not be measured due to the interference from PAC. On completion, the cultures were centrifuged at 6000 rpm to separate the aqueous phase. The 2PE was recovered from the centrifuged biomass and PAC with methanol.

### Semi‐continuous cultures

In the semi‐continuous experiment, the yeast was cultured in a 50 mL tube with working volume of 15 mL, using SGJ media. The tubes were sterilised and capped with a sterile sponge, and cultured at pH 4, at 20 °C. Two types of fermentation were examined; in the first the media was removed and the contained biomass discarded. In the second the media was centrifuged at 5000 rpm for 5 min and the resulting biomass pellet returned to the shake flask. The tube was placed in a 2.5‐inch cup and shaken at 150 rpm, providing higher shaking and air transfer. The supernatant was removed and replaced according to the calculation of dilution ratio 1/3, 1/6, 1/9 and 1/12 or mean retention time (MRT) 3, 6, 9 and 12 days.

$$\text{Dilution ratio}\;\left(\mathrm{da}y^{-1}\right)=\frac{\dot{V}}{V_{0}}\;\left(\mathrm{da}y^{-1}\right)$$

$$\text{Mean Retention Time}\;\left(\mathrm{day}\right)=\frac{1}{\text{Dilution ratio}}=\frac{V_{0}}{\dot{V}}$$

Therefore,

$$\dot{V}=V_{0}\times \text{Dilution ratio}=\frac{V_{0}}{\mathrm{MRT}}$$

where $\dot{V}\dot{=\text{volume taken out}\;\mathrm{per}\;\mathrm{day}\;\left(L\;\mathrm{da}y^{-1}\right)\;}$

$$V_{0}=\text{working volume of tube}\;\left(L\right)$$

### Use of absorbents for increased production of 2PE

*In situ* liquid–liquid adsorption was undertaken with oleyl alcohol (OA) and dodecane (DDC). The toxicity of both solvents to *M. pulcherrima* was studied in 25 mL culture in 125 mL shaking flasks, cultured at 20 °C for 15 days. The culture was conducted in SGJ media with added L‐phenylalanine at 2 g L^−1^ as a sole nitrogen source. The solvent toxicity was determined by the level yeast cell mass.

The 2PE adsorption capacity of the powdered activated carbon was calculated by adding 0.5 g of adsorbent to 10 mL aqueous 2PE solution (1000 mg L^−1^ concentration). The mixture was agitated at 20 °C for 3 days. The remaining 2PE concentration in the aqueous phase was analysed by HPLC‐UV. Langmuir adsorption characteristics and the maximum capacity was calculated by using activated carbon 0.10 g in aqueous 2PE concentration from 10, 20, 40, 60, 80 and 100 μL in 10 mL distilled water. 2PE adsorption equilibrium in solvents were conducted. PAC 0.1 g and 2PE 10.0 μL were added to 10 mL of distilled water and shaken at 150 rpm, 20 °C overnight. Residual 2PE in aqueous phase was determined by HPLC‐UV.

### Culturing M. pulcherrima in a batch 2 L controlled bioreactor

**Figure 1:**
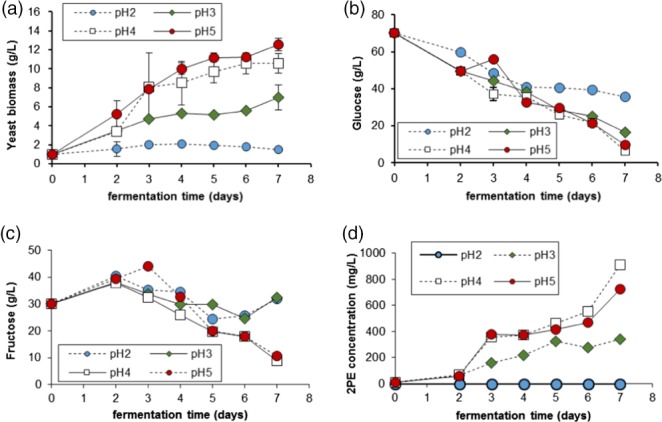
Effect of pH on: (a) yeast biomass production; (b) glucose catabolism; (c) fructose consumption; and (d) 2‐phenylethanol in M. pulcherrima cultured in SGJ media at 20 °C, error bars are given as one standard deviation (n = 3).

Experiments in bioreactors were conducted in 2 L Fermac 320 Bioreactor Fermenter (Electrolab Biolab Ltd). Temperature, pH and dissolved oxygen (DO) were controlled through the controller system using probes and were kept constant (Fig. 1(a)). The threshold for automatic control was pH ± 0.05, temperature ± 0.1 °C and DO ±1 unit. The bioreactor jar was sterilised prior to fermentation through autoclaving at 121 °C for 15 min. The study of aeration rate in *de novo* 2PE production from *M. pulcherrima* was carried out by fixing the air inlet flow rate to the bioreactor. The aeration was fixed during the experiment at 0.5, 1 and 2 L min, with working volume 2 L in the bioreactor. The maximum aeration rate of 2 L min^‐1^ represented 1 working volume/min in the bioreactor. The conditions of fermentation were taken from the optimised shake flask experiments and were set at pH 4, 20 °C with an agitation speed of 180 rpm. The bioreactor jar and lid were autoclaved prior to fermentation.

### Continuous de novo 2PE production

In the continuous bioreactor experiment, the input and output flow were controlled through a peristaltic pump (Fig. 1(b)). The input flow rate $\dot{V}$ was calculated in the same manner as for the semi‐continuous mode and controlled by the feed peristaltic pump. The overflow output was taken by a separate peristaltic pump at the top of the fermenter. The experiment was started in batch mode for 7 days and continued in continuous mode from day 8. During the continuous period, sterile fresh SGJ media was fed into the bioreactor by the peristaltic pump. The flow rate was controlled by an on/off switch operated in a cycle (10 min per cycle) regulated by the main controller. The condition was controlled at 20 °C automatically and pH 4 was maintained by adding HCl 1 mol L^−1^ and NaOH 1 mol L^−1^, automatically, regulated by the main controller. The continuous phase was carried on with dilution rate of 8 h or 1/3 d^‐1^ (mean cell retention time, MRT 3 days) as this should provide the mean biomass retention time 3 days in the chemostat, equating to approximately the span of growth period in batch fermentations.

### Batch ex novo production in a 2 L controlled bioreactor

*Metschnikowia pulcherrima* was cultured in the bioreactor with activated carbon as an *in situ* extractant and controlled in batch mode as described above. The media for bioconversion was SGJ media with the addition of 20 g L^−1^ of L‐phenylalanine. Activated carbon (GAC2040, 20–40 mesh) was sterilised and added directly in the bioreactor in steps of 20 g or 50 g when 2PE in the aqueous phase was over 500 mg L^−1^. The threshold of 500 mg L^−1^ was selected as the requirement for adding new activated carbon. The maximum activated carbon was 150 g.

### Continuous ex novo 2PE production

Fermentation in the bioreactor was started in batch mode similar to the previous study and continuous mode was started on day 3. To conduct continuous mode, the input feed was fed directly into the bioreactor and the output was via a cross‐flow membrane (MiniKros Sampler Filter Module, Spectrum Labs Ltd) with pore size of 750 kD and ID 1.0 mm) to separate the biomass from the output stream allowing the aqueous phase, with 2PE, to pass through and the yeast‐rich retentate to be returned to the bioreactor. The biomass in the bioreactor was controlled by direct removal to maintain a steady concentration. The aquoues permeate was then passed through an adsorption column with GAC2040 (20–40 mesh) as the adsorbent. 2PE was removed by the adsorbent and the rest of the stream sent to waste. When the adsorption in the column was at its limit, methanol was used as the solvent to leach 2PE from the column.

### Analytical methods

Yeast biomass was calculated gravimetrically by weighing the freeze dried samples (∼0.1 g). Arabitol, glucose and fructose were determined on a Shimadzu 10AVP HPLC system (Shimadzu Corp., Japan) fitted with a pump (LC‐10 AD), an auto injector (SIL‐10 AD) and a system controller (SCL‐10A). Filtered (0.22 μm, Millipore, UK) hydrolysate samples (10 μL) were injected without dilution into a 300 × 7.8 mm Aminex HPX‐87H column (BioRad, CA, USA) at 65 °C fitted with RID‐10A detector. Isocratic elution took place over a 25 min period at 0.6 mL min^‐1^ using 0.2 μm‐filtered and degassed 5 mmol L^−1^ sulfuric acid. 2‐phenylethanol content was determined using an identical HPLC system with a UV detector, filtered samples diluted by a factor of 20 and 10 uL injected onto a Dionex column eluted at 40:60 water:acetonitrile at 0.4 mL min^‐1^. The concentrations were calculated against known standards. Arabitol was confirmed by HPLC‐ MS (Bruker MicrOTOF electrospray time‐of‐flight mass spectrometer (ESI‐TOF), coupled to an agilent high performance liquid chromatography (HPLC) unit) and standardised against an arabitol solution 1 g L^−1^ (diluted 20 times) compared with ribitol and xylitol solutions.

## RESULTS AND DISCUSSION

### Optimisation of de novo 2‐PE production

*Metschnikowia pulcherrima* was cultured in shake flasks to optimise the conditions for 2PE production (Fig. 1). The yeast was cultured in a synthetic grape juice (SGJ) to mimic the wine must in which *M. pulcherrima* has previously demonstrated moderate 2PE production.^8^ *Metschnikowia pulcherrima* is known to survive at low pH,^20^ however at pH 2 the growth rate was very low; while some growth was observed after 24 h, all growth had ceased by 72 h and decreased after this point. At first, both fructose and glucose were consumed at a similar rate, although after 5 days no further sugars were metabolised at this pH. At higher pH, the amount of biomass was increased substantially with the optimal being pH 5. The yeast reached stationary phase after approximately 72 h, although this did depend on the pH. The growth rate of *M. pulcherrima* was highest at pH 4 and pH 5 with a maximum growth rate at pH 4 of 1.04 g g^‐1^ d^‐1^ and 1.03 g g^‐1^ d^‐1^ for pH 5. This was not only a reasonable rate for biomass production but, helpfully, pH 4 would ward off invasive species while still guaranteeing high productivity from the yeast.

Hexose sugars such as glucose, fructose and mannose are the common and preferred carbon source for most yeast species, and it is clear that *M. pulcherrima* is well adapted to using them.^22^, ^23^ In this media, *M. pulcherrima* showed a preference for glucose over fructose, with less than 20 g left in the supernatant after fermentation at pH 3 to pH 5. Fructose was also mostly consumed at higher pH values throughout the experiment, albeit at a lower rate than glucose, but a sudden halt was observed in the early stationary phase in the most acidic media. This suggests that very low pH is detrimental to the metabolism of *M. pulcherrima,* even though the yeast still survives. 2PE production was examined over the pH range (Fig. 1(d)) and follows a similar trend to the biomass production, with less acidic media being more favourable to the production of 2PE. This was expected as the microbial production of 2PE has mainly been observed in the exponential phase and thus should correlate with biomass production,^24^, ^25^ though higher values were observed at pH 4 than at pH 5 suggesting that the production per cell is optimal at pH 4. Surprisingly, *M. pulcherrima* was able to produce up to 1 g L^−1^ of 2PE, a value higher than reported for batch fermentation for any other species of yeast and an order higher than *M. pulcherrima* was reported to produce in wine fermentation.^8^

**Figure 2:**
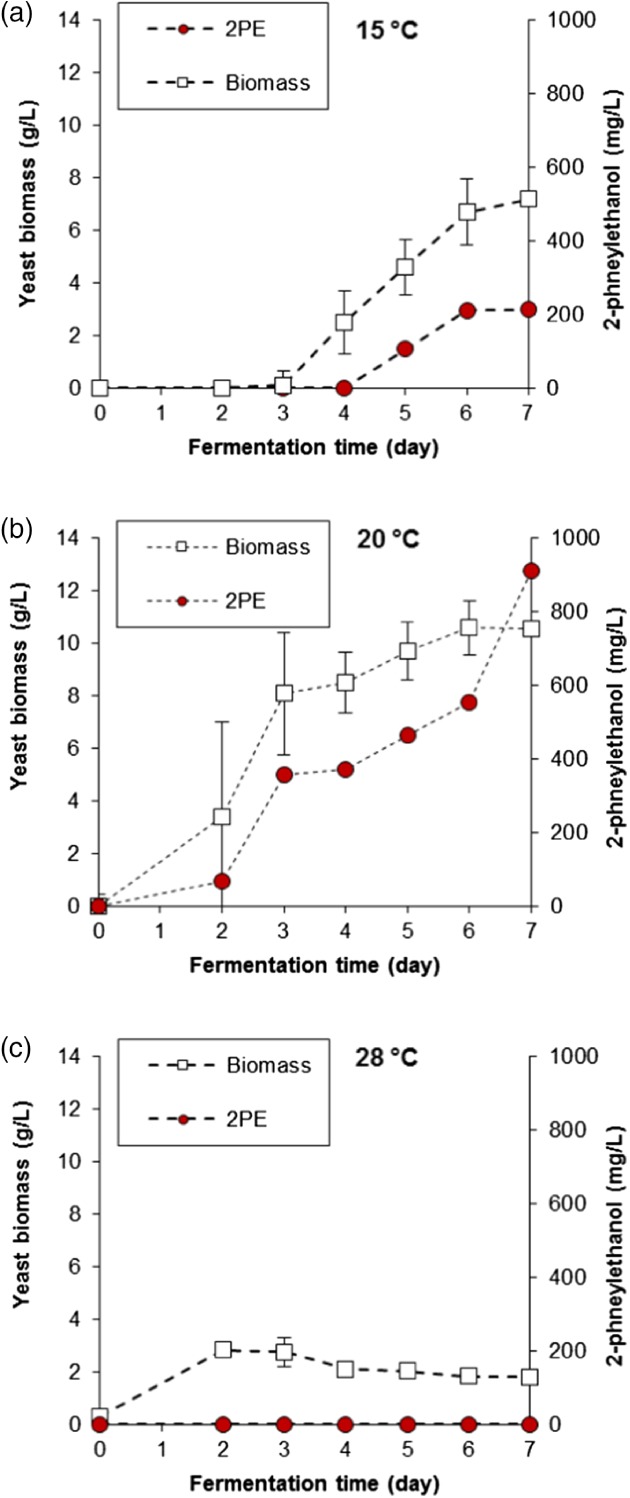
Fermentation profiles of M. pulcherrima culture in shake flasks in SGJ media pH 4 at 15 °C, 20 °C and 28 °C (n = 3, error bars represent one s.d.).

Temperature was also optimised, testing with yeast cultured at 15 °C, 20 °C and 28 °C (Fig. 2). Meso‐psychrophilic behaviour of *M. pulcherrima* is evident, with maximum yeast biomass achieved at 20 °C, followed by a slight decrease at colder conditions and barely any growth at 28 °C. This performance was also mirrored by the related sugar consumption, with a sudden halt in carbon absorption after 3 days when the strain was grown at the highest temperature (data not shown). In this regard, *M. pulcherrima* differentiates itself from other non‐*Saccharomyces* yeasts that usually prefer a temperature around 28 °C. This is also true of *Kluyveromyces marxianus*, another yeast reported to be a good 2PE producer, which can sustain a high growth rate at around 40 °C.^26^ A lower biomass growth was accompanied by lower 2PE yields, with only around 200 mg L^−1^ been produced at 15 °C and none detected at 28 °C.

**Figure 3:**
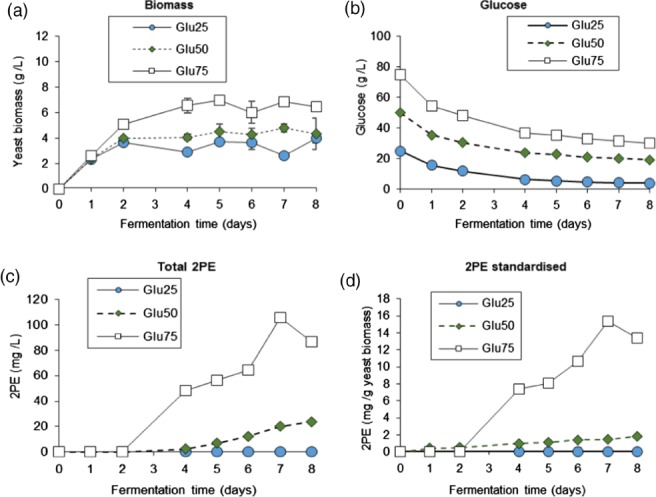
Effect of glucose concentration on: (a) biomass production; (b) glucose uptake; (c) 2‐phenyl ethanol production; and (d) arabitol production for M. pulcherrima cultured at 20 °C, pH 4, in modified SGJ, with no fructose and varying glucose concentrations of 25 g L^−1^ (Glu25), 50 g L^−1^ (Glu50) and 75 g L^−1^ (Glu75). The C/N ratio was kept constant for all glucose loadings.

Up to this point a relatively high sugar loading (100 g L^−1^ of total sugar) was used. However, sugar loading can have a strong effect on the formation of products due to metabolic and osmotic stresses. To understand how sugar concentration affected the 2PE production in *M. pulcherrima*, the yeast was cultured in a modified SGJ media at 20 °C and pH 4. Glucose was used as the sole carbon source. The glucose concentrations examined were 25, 50, and 75 g L^−1^ (Fig. 3). The C/N ratio was kept constant for all the media used in order to negate the effect of having a different C/N ratio that could affect the results at lower carbon loadings. The lower the glucose concentration, the lower the growth, with the highest yeast biomass achieved with 75 g L^−1^ of glucose. This demonstrates that there is no osmotic inhibition, and that under these conditions *M. pulcherrima* could be described as an osmophilic yeast. This is potentially beneficial as being osmophilic means that the yeast is less prone to contamination and could grow in non‐sterile conditions, as well as reducing the volume of the bioreactors needed to sustain production.

The concentration of glucose also had a strong effect on the 2PE production of *M. pulcherrima* (Fig. 3(c)). 2PE production from *M. pulcherrima* is highest in SGJ media with glucose at 75 g L^−1^, while some 2PE was observed at lower sugar loadings, none was observed at a glucose loading of 25 g L^−1^.

**Figure 4:**
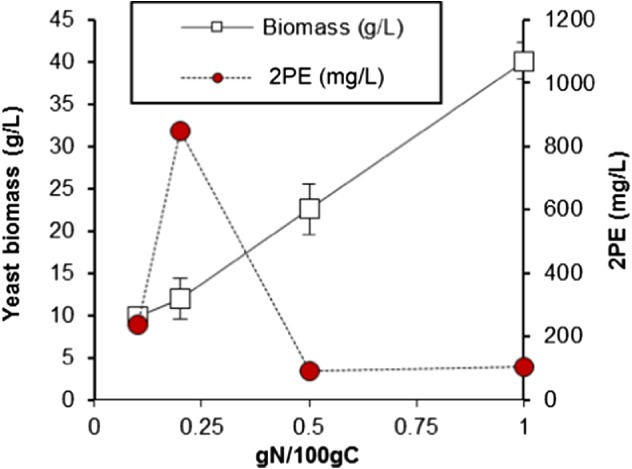
Effect of N/C ratio on growth and production of 2PE and in SGJ media with no fructose at 20 °C, pH 4 over 7 days, error bars are given as one standard deviation (n = 3).

In yeast, 2PE is produced *de novo* through the sequential action of the Shikimate and Erhlich pathways. In the Shikimate pathway, L‐phenylalanine (along with other aromatic amino acids) is synthesised from glucose; it is then catabolised via the Erhlich pathway to produce 2PE. Such pathways enable yeast to acquire nitrogen from amino acids when a preferred nitrogen source, such as ammonium, is not available. Importantly, both the source of nitrogen and its concentration have been reported to promote the activation or suppression of metabolic pathways,^27^ and influence the production of 2PE,^28^ through a phenomenon known as nitrogen catabolite repression. Here, genes encoding enzymes involved in metabolising non‐preferred nitrogen sources, such as phenyalanine, are not expressed when preferred nitrogen sources, such as ammonium, are in abundant supply. However, when ammonium is limited these genes are switched on, thus activating metabolic pathways that enable the yeast to utilise alternative nitrogen sources. Consequently, the effect of a range of concentrations of (NH_4_)_2_PO_4_ as the sole nitrogen source were examined (Fig. 4). As expected, a lower C:N ratio resulted in a higher final biomass, however, 2PE production was highest at a concentration of 0.2 g N per 100 g C. Therefore, despite correlating biomass with 2PE production, this data suggests that ammonia actively inhibits 2PE production in *M. pulcherrima*, as previously reported for other yeast species.^28^, ^29^ The presence of specific nitrogen sources, including ammonia, may regulate both the Ehrlich and Shikimate pathways in a number of places. For instance, *ARO8* and *ARO10* are two key genes in the Ehrlich pathway and their expression has been shown to influence 2PE production^30^ and to be repressed by ammonia, resulting in lower 2PE production.^29^

### Semi continuous fed‐batch culturing

Metschnikowia pulcherrima is highly capable of producing 2PE through the de novo conversion of glucose in a batch mode in shake flasks. The compound was produced mostly during the growth phase. However, in batch mode there is a significant lag phase, where the yeast is acclimatising to the conditions and media. This is unavoidable under these operating conditions although it can be potentially removed by producing the 2PE in a semi‐continuous or continuous mode instead. This would also benefit from being able to produce 2PE in smaller reactor volumes with less down time of the bioreactor.

To assess the suitability of M. pulcherrima for this production mode, the broth in the fermentation was partially removed periodically followed by the addition of fresh new media. The yeast can then use the nutrients in the fresh media to generate new biomass, replacing what was previously removed in a rapid timeframe with no lag time.^31^ This is similar to using a continuous reactor on dilution ration or mean cell residence time (MRT) which can be controlled by how much the replaced and removed volume is applied to the fermentation, similar to the feeding flow rate of a continuous process.

This semi‐continuous experiment was conducted using a 50 mL tube with working volume of 15 mL, with similar ratio of media to air. The tubes were sterilised and capped with a sterile sponge for effective gas transfer. Two types of fermentation were examined. In the first fermentation, the aliquot of media removed contained biomass which was then discarded. This was compared with the second fermentation in which the biomass was not discarded but retained in the vessel. This was achieved by centrifugation of the removed supernatant and removal of the biomass pellet. The culture tubes were incubated at 20 °C with SGJ media at pH 4 with tartaric acid 7 g L^−1^ as a pH buffer and internal standard. The supernatant was removed and replaced according to the calculation of dilution ratio 1/3, 1/6, 1/9 and 1/12 or mean retention time (MRT) 3, 6, 9 and 12 days.

**Figure 5:**
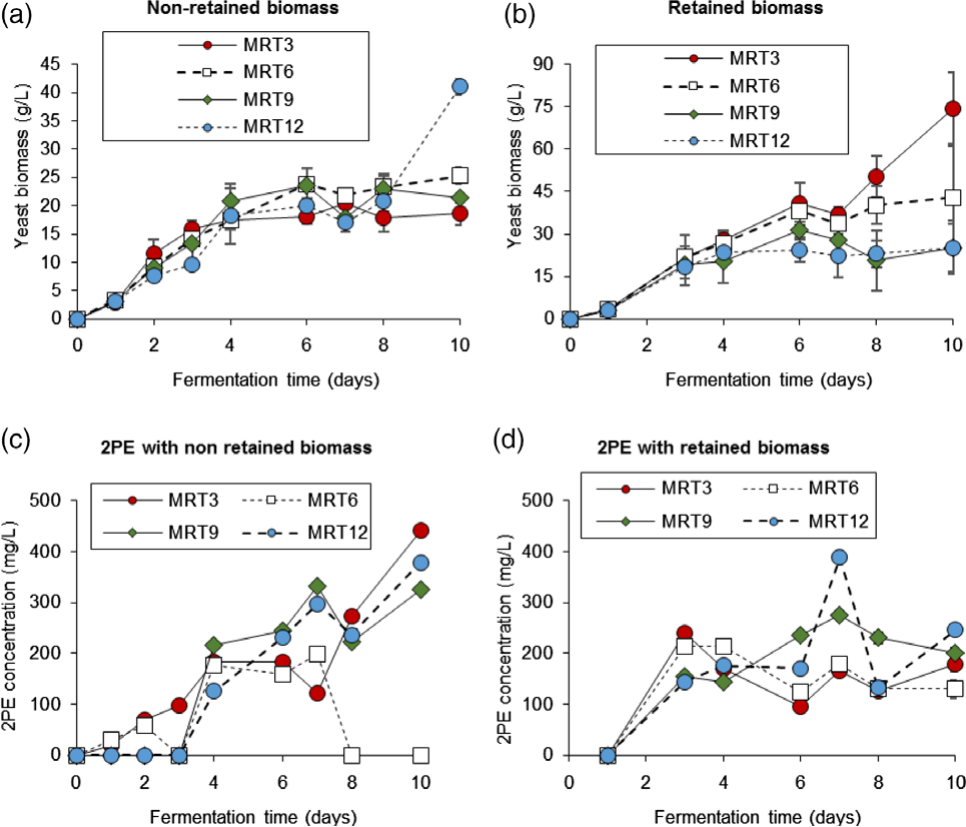
Growth, 2PE and sugar utilisation in semi‐continuous shake flask culture of M. pulcherrima with mean retention time (MRT) 3, 6, 9, 12 days (dilution rate 1/3, 1/6, 1/9 and 1/12 day^‐1^, respectively). Semi‐continuous process started on day 3 (n = 3, error bars represent one s.d.).

The biomass and 2PE production from M. pulcherrima in semi‐continuous culture is shown in Fig. 5. The culture with higher dilution ratio saw the greatest biomass production, presumably as there was more fresh media available. Therefore, a dilution ration of 1/3 day^‐1^ (the culture taken out was 1/3 of the working volume each day and was replaced with fresh media to the same volume) had the higher growth in the stationary period. Less biomass was achieved in 1/6, 1/9 and 1/12 day^‐1^, respectively. Naturally, the biomass in the retained system is higher than in the culture without the retained biomass process. With the retained biomass system, the highest biomass achieved was close to 75 g L^−1^ after 10 days which is approximately 6–12 times the usual biomass concentration achieved in any other cultures. The retained system could possibly be achieved on a larger scale by letting the culture settle before broth replacement, although this higher biomass loading would likely reduce the oxygen transfer and mixing degree of the culture.

The yeast in both systems seemed to produce 2PE in similar amounts, approximately 170–250 mg L^−1^, and there was no significant difference between dilution ratios. The unusually low 2PE concentration may have resulted from the higher aeration rate in the shaking 50 mL tube. This also contributed to the higher‐than‐usual biomass production in the non‐retained biomass culture as the higher aeration supplied more oxygen in the metabolism which yields more ATP for new cell generation but less 2PE production. The higher metabolism rate was also seen in the glucose uptake rate in both systems where the glucose was used so rapidly that none was left in the culture by the end of the day. These preliminary experiments demonstrated that 2PE can be produced in a semi‐continuous culture, although oxygen demands are likely to be key to higher production.

### Ex novo production of 2PE in shakeflasks

**Figure 6:**
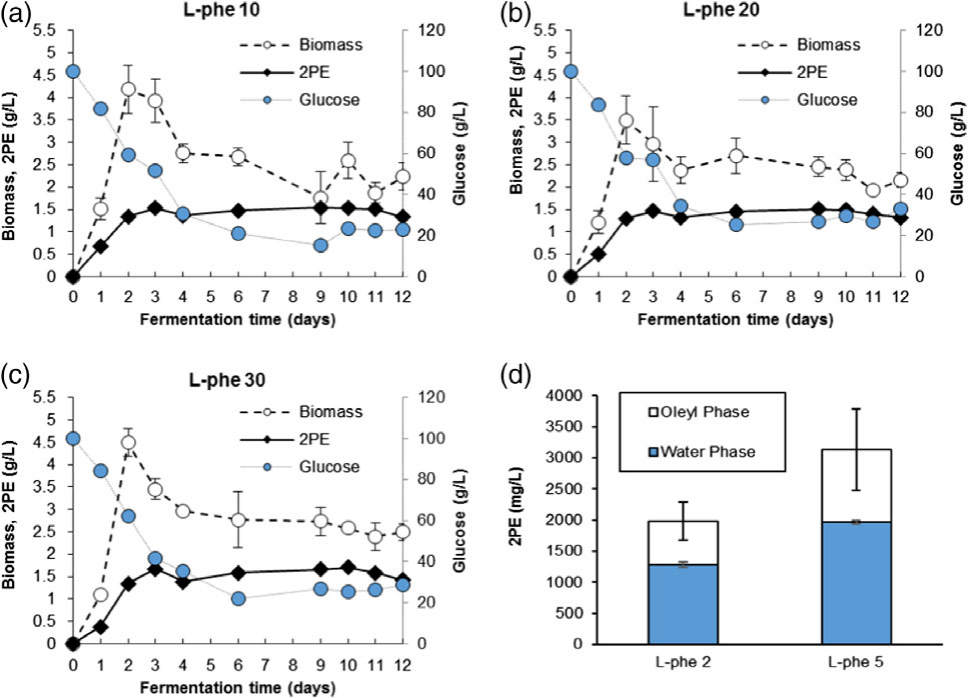
Bioconversion of phenylalanine to 2PE with (a) 10 g L^−1^ phenylalanine, (b) 20 g L^−1^ phenylalanine, (c) 30 g L^−1^ phenylalanine. (d) Liquid extraction of 2PE from cultures using oleyl alcohol, error bars are given as one standard deviation (n = 3).

The addition of exogenous phenylalanine to the growth medium has been shown to lead to increased levels of 2PE due to *ex novo* conversion.^12^ To test this with *M. pulcherrima*, the yeast was cultured at 20 °C, pH 4 over 12 days in SGJ media with additional L‐phenylalanine (10, 20 and 30 g L^−1^) containing no other additional nitrogen sources (Fig. 6). The yeast biomass achieved was similar irrespective of the phenylalanine loading, but was substantially lower than that without phenylalanine. In all the cultures yeast biomass generally reduced after 3 days in the stationary phase, down to approximately 2.5 g L^−1^. Ethanol was also produced in the cultures, having a final concentration of 4.6, 3.6 and 3.7 g L^−1^, for cultures with 10, 20 and 30 g L^−1^ phenylalanine, respectively. Ethanol has a minor synergistic inhibitory effect with 2PE (see Supporting information). It is probable that, with this additional loading of ethanol and high concentration of 2PE, the biomass production was inhibited to some extent.

The yeast started to produce 2PE from the first 24 h for all the cultures, irrespective of phenylalanine concentration. For all cultures production reached a maximum during day 3 before stagnating. The maximum production of 2PE for all cultures was between 1520 and 1710 mg L^−1^, which is the approximate toxicity limit of 2PE, even without ethanol present. Similarly, glucose was used rapidly from the start of culture, and even though the yeast biomass stopped increasing after day 2, the glucose utilisation continued until day 4, although as 2PE concentration increased the uptake completely stopped after that point. This indicates that *M. pulcherrima* can convert L‐phenylalanine to 2PE effectively, up to the 2PE threshold it can tolerate.

In order to produce higher volumes of 2PE by biosynthesis, 2PE concentration must be kept under this toxic threshold. To achieve this, continual liquid extraction was attempted, removing the 2PE formed continually from the reaction into a different phase away from the yeast. One of the major difficulties in using *in situ* extraction is that the extractant must have a high affinity to 2PE while not affecting the growth of the yeast or the 2PE biosynthesis in the yeast. To this end, two solvents were selected for screening liquid–liquid *in situ* 2PE extraction in the biosynthesis; oleyl alcohol (OA) and dodecane (see Supporting information); OA at 20% was demonstrated to be optimal.

*In situ* extraction with oleyl alcohol in 2PE biosynthesis in *M. pulcherrima* was determined with the addition of 2 and 5 g L^−1^‐phenylalanine in SGJ media with 5 mL oleyl alcohol in 25 mL *M. pulcherrima* culture (Fig. 6(d)). 2PE concentrations in the culture in both the aqueous supernatant and oleyl alcohol phase were analysed. The two phases mixed to some degree during shaking in the flasks, presumably allowing reasonable product transfer, but separated easily when left to settle for 30 min.

2PE production was greatly improved when using oleyl alcohol as an *in situ* solvent; for example, on addition of 2 g L^−1^ of phenylalanine, a yield of 1280 mg L^−1^ in the aqueous phase and an overall yield of 1985 mg L^−1^ were observed. Similarly, with 5 g L^−1^ L‐phenylalanine added to the culture, a 2PE yield of 1961 mg L^−1^ in the aqueous phase and 3133 mg L^−1^ overall was achieved.

The 2PE produced in both cultures was approximately similar to the theoretical 2PE percentage conversion from L‐phenylalanine, given as approximately 74% by weight.^9^ This suggests that most of the L‐phenylalanine added was converted to 2PE, as well as further glucose being funnelled into the *de novo* synthesis. Similar results with oleyl alcohol as an *in situ* extractant were achieved by Etschmann *et al*. using *K. marxianus* in 70 mL molasses medium with 7 g L^−1^ L‐phenylalanine and 30 mL oleyl alcohol.^14^ This yielded a total 2PE of 3 g L^−1^, increased from 0.8 g L^−1^ without the extractant phase.

The use of a non‐toxic solvent in the yeast culture to remove 2PE was highly effective. However, the affinity of 2PE to oleyl alcohol is useful in removing the compound from the aqueous phase but raises further technical issues in separating the 2PE from the oleyl alcohol phase after fermentation. A typical liquid–liquid extraction usually uses a solvent with a low boiling point that can easily be separated from the desired compound by solvent evaporation. However, oleyl alcohol (C_18_H_36_O) has a boiling point of 330–360 °C while 2PE has a boiling point at 220 °C. The high boiling point of oleyl alcohol, therefore, makes the separation of 2PE from oleyl alcohol difficult and uneconomical.

**Figure 7:**
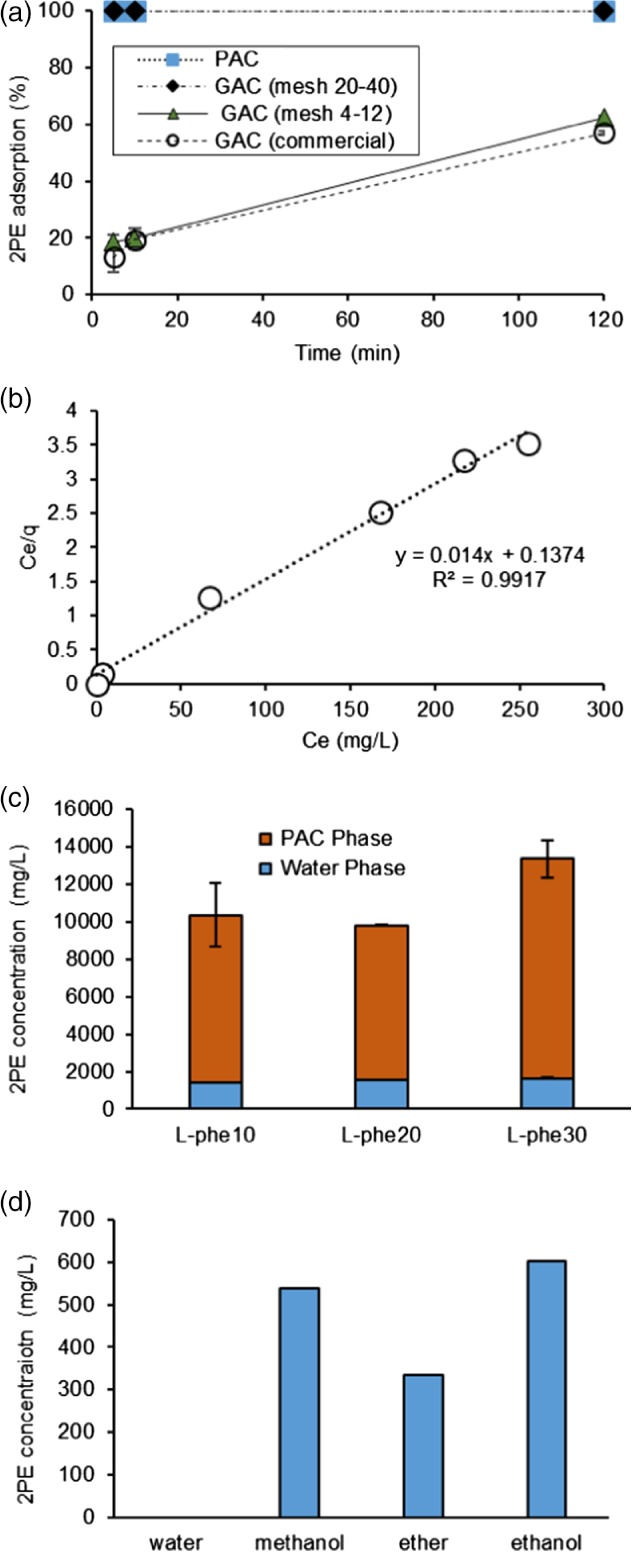
Solid extraction of 2PE using activated carbon: (a) the sizing; (b) Langmuir isotherm plot of 2PE adsorption on powdered activated carbon (PAC). (c) 2PE production by biosynthesis from L‐phenylalanine with powdered activated carbon (PAC) as an in situ extractant in M. pulcherrima culture 25 mL in SGJ media pH 4 at 20 °C after 7 days (n = 3). (d) 2PE concentrations at equilibrium in different solvents with 2PE 10 mg and PAC 10 mg in 10 mL of solvents in leachability study.

Due to the ease of recovery, solid phase adsorbents may provide a better choice for 2PE recovery overall, as the target compound can be recovered from the adsorbent easily with a suitable low boiling point solvent which can be removed by distillation. Activated carbon is a highly porous material with a large surface area, typically more than 1000 m^2^ g^‐1^. The extensive surface area gives the material high adsorption capacity. In addition, activated carbon is a non‐toxic material and widely used in adsorption applications for plant, animal and human use. The adsorbent was used in four different sizes, powdered, granular Draco 20–40 mesh, granular Draco 4–12 mesh, and a granular commercial activated carbon. The adsorption study used an initial 2PE aqueous solution of 1000 mg L^−1^ and added activated carbon 1 g per 50 mL solution in 125 mL Erlenmeyer flasks, shaking at 20 °C and 180 rpm. The experiments were done in triplicate for each size. The samples were collected from the aqueous phase after 5 min, 10 min and 2 h and analysed for 2PE concentration (Fig. 7(a)).

PAC and the small size granular activated carbon GAC 2040 (20–40 mesh) performed well in the 2PE adsorption test, with 2PE being completely adsorbed within 5 min. This suggests that GAC2040 or PAC would be suitable for this process. The adsorption rate is slower in the larger size granular activated carbon GAC0412 (4–12 mesh). It only adsorbed 20% of the 2PE in the first 5 min and only reached 62% after 2 h. The adsorption of 2PE on the commercial GAC, which has a size similar to GAC0412, was slightly below this.

The 2PE adsorption characteristic and isotherm equilibrium for PAC was further examined by varying the concentration of 2PE aqueous solutions with 0.1 g of adsorbent. 2PE in the aqueous‐phase (C_e_) and in the adsorbed‐phase (q_e_) were then calculated (Fig. 7(b)). The adsorption was determined using the Langmuir isotherm, plotting the C_e_/q_e_ and C_e_ according to this correlation. The 2PE adsorption on PAC fits the Langmuir isotherm (r^2^ = 0.99). The maximum Langmuir adsorption capacity (q_max_) was therefore 0.807 g g^‐1^ of 2PE.

Due to the excellent 2PE absorption demonstrated by PAC, the adsorbent was then added to cultures of *M. pulcherrima*. The cultures were grown on 25 mL SGJ media with L‐phenylalanine 10, 20 and 30 g L^−1^ and 0.5 g PAC added. The culture was incubated at 20 °C for 7 days. Biomass could not be measured due to the interference from PAC. 2PE production by *M. pulcherrima* with PAC was demonstrated to be increased dramatically (Fig. 7(c)). 2PE in the aqueous phase was approximately 1500 mg L^−1^ in all L‐phenylalanine concentrations, similar to the previous experiments. However, 2PE in the solid phase which was adsorbed on PAC increased greatly, being the equivalent of 7 to 10 g L^−1^. In the culture with 10 g L^−1^ L‐phenylalanine, 2PE was produced at 7.4 g L^−1^, showing that the yield corresponded exactly to the theoretical yield (theoretical yield of 2PE production 0.74 g g^‐1^ L‐phe), suggesting *M. pulcherrima* can convert all L‐phenylalanine to 2PE.

In the *M. pulcherrima* culture with L‐phenylalanine at 20 and 30 g L^−1^, the 2PE produced was not increased by the increasing amount of precursor, with yields of only 0.332 and 0.337 g g^‐1^ phenylalanine, respectively. This suggests that PAC adsorbed 2PE to its full capacity under these culture conditions, with the excess 2PE production potentially inhibiting further 2PE conversion. For practical purposes, a rough estimate of 0.5 g PAC may be suggested for each 10 g L‐phenylalanine added in the biosynthesis.

*Metschnikowia pulcherrima* with activated carbon as an *in situ* adsorbent was shown to be an excellent 2PE production system for bioconversion from L‐phenylalanine; a maximum of 14 g 2PE L^−1^ was demonstrated. This is higher than 2PE production from other yeast systems presented in the literature using the adsorbants Hytrel, polypropylene glycol 1200, or polystyrene resin.^14^, ^15^

The 2PE can be recovered from the solid adsorbant through extraction with a suitable solvent followed by evaporating the solvent, yielding purified 2PE. Therefore, the selection of solvents is vital to the 2PE biosynthesis from L‐phenylalanine. The ideal solvent should show the highest 2PE affinity and have the lowest boiling point. The 2PE affinity of solvents was investigated by the adsorption of 2PE where 2PE (10 mg) and PAC (0.1 g) were added to the solvents (10 mL).

2PE can be extracted slightly more effectively by ethanol than methanol (53%) and far better than diethyl ether (33.5%) under the conditions tested and would be a suitable solvent for recovery of the target compound (Fig. 7(d)). While the methanol fraction predominantly contained 2PE, other polar components were also observed in this phase, including ethanol, tartaric acid, malic acid and arabitol. The 2PE would therefore need to be purified by distillation prior to being sold.

### Scale‐up of the de novo and ex novo cultures of M. pulcherrima to 2 L controlled stirred bioreactors

Following these promising optimisation results, *M. pulcherrima* was scaled up to produce 2PE in a controlled stirred bioreactor. The pH, dissolved oxygen (DO) and temperature were all controlled automatically and logged continually. Initially, the effect of aeration, which can be controlled more accurately in the bioreactors, was examined for the *de novo* synthesis of 2PE. Three different aeration rates (0.25:1, 0.5:1 and 1:1 v/v) were applied and the culture conditions were chosen according to the optimisation given above (pH 4, 20 °C, 180 rpm).

**Figure 8:**
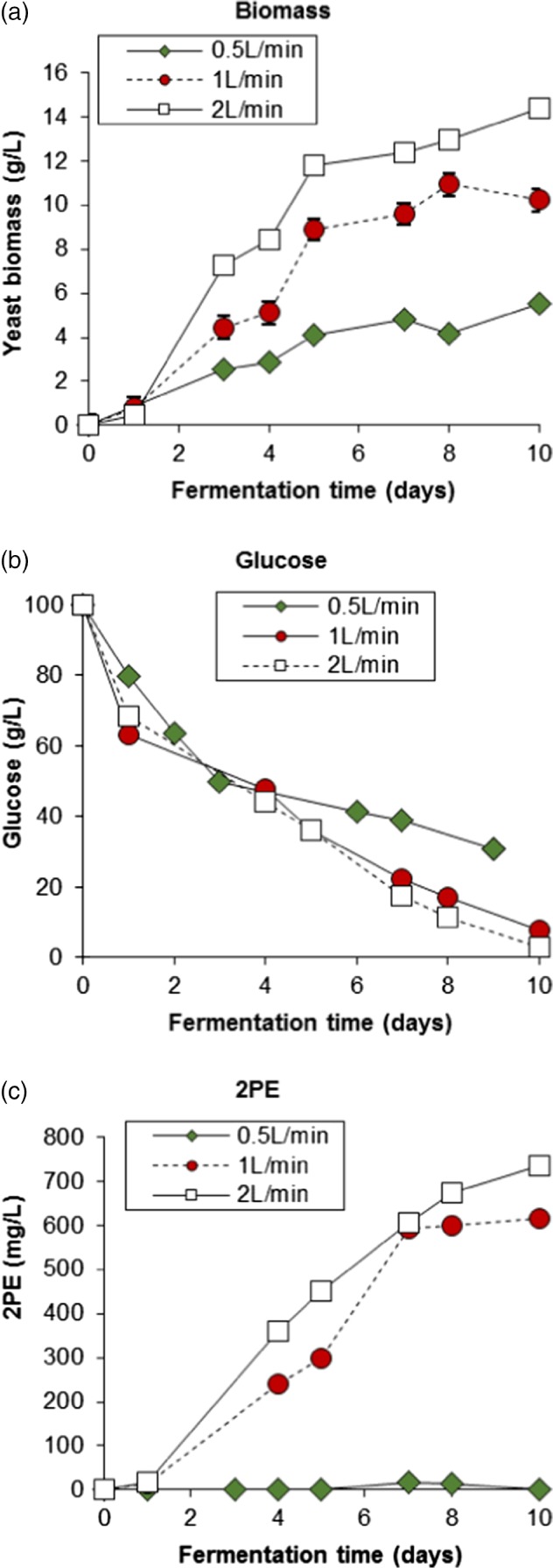
Effect of aeration on the yeast biomass, 2PE and glucose catabolism for M. pulcherrima culture grown in SGJ media at pH 4, 20 °C in 2 L bioreactors.

The fermentation profile of the conditions in the bioreactor during the different aeration rates are given in the Supplementary information. The conditions in the bioreactor such as pH and temperature were well controlled and remained at pH 4 and 20 °C throughout the experiment for all aeration conditions. The DO dropped substantially during the growth phase of the yeast during the experiments with 1 and 2 L min^‐1^ airflow in the first few days of the fermentation, and remained at approximately 50% after this point. The DO in the fermenter with 0.5 L min^‐1^ remained at 100% throughout the fermentation (see Supporting information). The biomass and 2PE productivity achieved in this bioreactor were similar to the shake flasks, with up to 14 g L^−1^ of yeast biomass and 700 mg L^−1^ of 2PE (Fig. 8). The biomass and 2PE production increased in more aerated conditions, with 6 g L^−1^ of yeast and only trace amounts of 2PE produced at 0.5 L min^‐1^, increasing to 14 g L^−1^ and 700 mg L^−1^, respectively, when the air flow was taken to 2 L min^‐1^.

Despite having simple control over culture conditions, batch operations in bioreactors also show drawbacks such as a long lag time and accumulation of toxic compounds, as well as an inhibitory effect of 2PE in *Candida spp* and related strains, among which *M. pulcherrima* is reported.^32^ The production of 2PE is related to the activity of the Shikimate pathway, which converts glucose to L‐phenylalanine, and the Ehrlich pathway, which produces 2PE from L‐phenylalanine, during exponential growth, with the former probably the bottleneck of *de novo* 2PE production due to it's pivitol role in synthesising phenylalanine. Given this, moving to a continuous mode would delay the insurgence of the stationary phase, thus potentially increasing the overall yield.

**Figure 9:**
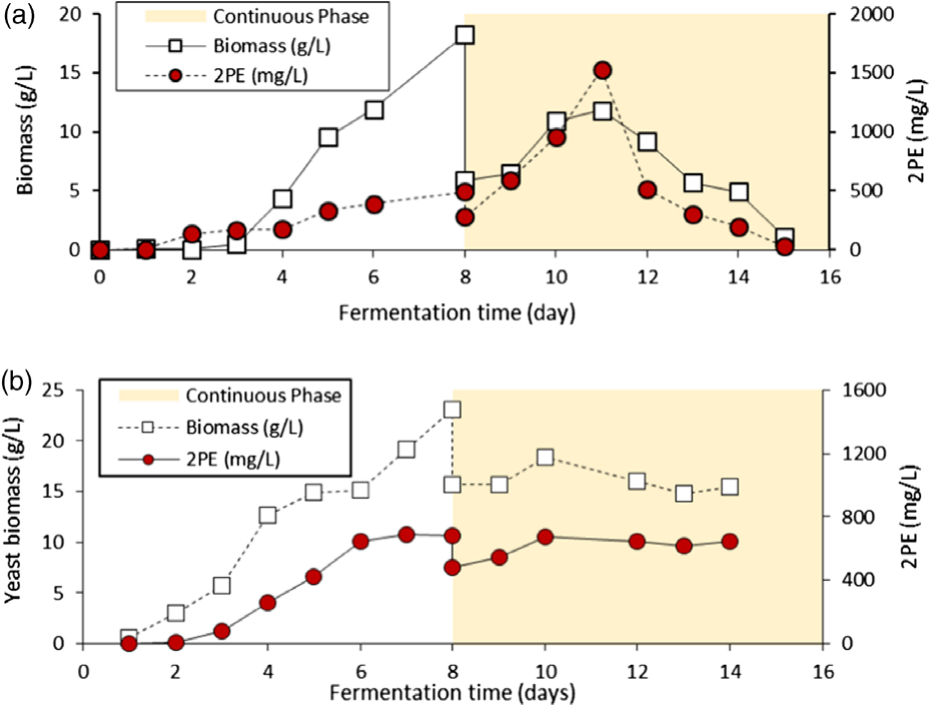
Continuous de novo M. pulcherrima fermentation with (a) dilution ratio 1/3 d^‐1^ (HRT 3 days) at 20 °C, pH 4, (b) dilution ratio 1/6 d^‐1^ (HRT 6 days) at 20 °C, pH 4. The bioreactor was run in batch mode from start to day 7 and continuous mode from day 8.

To this end, the culture was started in batch mode for an initial 7 days, before being run in continuous mode from day 8. During the continuous period, sterile fresh SGJ media was fed into the bioreactor. The continuous phase was carried out with a dilution rate of 8 h or 1/3 d^‐1^ as this should provide the mean biomass retention time 3 days in the chemostat, which was approximately the span of growth period in batch fermentation (Fig. 9(a)). After day 3 the DO dropped substantially demonstrating that the yeast had entered the exponential phase (see Supporting information).

*Metschnikowia pulcherrima* grew very well in SGJ media in the batch mode, with a maximum biomass close to 20 g L^−1^ while producing up to 500 mg L^−1^ 2PE by day 8. On day 8, fresh SGJ media was fed to the bioreactor and the fermentation started to run in continuous mode, with the overflow pumped out from the bioreactor to keep the volume constant. The performance of the reactor in continuous mode is shown in the yellow phase in the figure. The yeast biomass decreased by approximately a third in the first day of continuous running suggesting the biomass was washed out with the overflow output. The reduced ratio biomass tallies with the dilution ratio of 1/3 d^‐1^. The new yeast cells seemed unable to reproduce fast enough to replace the cells lost in the first day of the continuous mode. However, the biomass recovered after this point and increased to 12 g L^−1^ by day 11. After this point the culture appeared to crash, with only 1 g L^−1^ of yeast present in the bioreactor after day 15.

The 2PE level in the bioreactor was also affected by the dilution at first, but in the first 24 h of continuous running the yeast was producing 2PE and by day 9 almost 600 mg L^−1^ was present. As the yeast biomass started to increase, 2PE concentration rose as well. The 2PE concentration was then higher than during the batch period. The continuous regime also meant that signalling compounds and other inhibitors accumulate less in the bioreactor resulting in higher product formation. As a result, more 2PE was produced and the concentration was increased to over 1600 mg L^−1^. After 3 days in the continuous phase, the accumulated 2PE was presumably too high, with the limiting threshold of 1500 mg L^−1^ being reached (see Supporting information). This is presumably the cause of yeast death, explaining the rapid decrease of biomass concentration.

The continuous flow bioreactor was shown to increase 2PE production substantially in the batch process, and the yeast initially responded well to this mode of operation and the new media in the first few days. In the dilution ratio 1/3 of working volume per day, the mean yeast cell resident time in the bioreactor was 3 days and was shown to be ideal for 2PE production. However, when the dilution rate was high and the yeast was operated at a young mean age, it increased the 2PE too much to the inhibitory level for the yeast and caused flush out from day 11. Therefore, a lower dilution rate would be needed to retain a longer biomass retention time in the bioreactor, and create a more stable 2PE platform.

To improve the stability of the system *M. pulcherrima* was cultured in a similar batch mode over 7 days before being run in continuous mode with dilution rate 1/6 d^‐1^ thereafter (Fig. 9(b)). Initially, high biomass and 2PE production was also observed for the yeast in batch mode. When switching to continuous mode the yeast biomass and 2PE were reduced in the first day because of the outflow flux from the bioreactor, but the yeast recovered rapidly. The bioreactor became stable after starting the continuous phase and a reasonably constant 15 g L^−1^ of yeast biomass and 2PE production of 620–670 mg L^−1^ was maintained.

At the lower dilution rate with less new influx media, 2PE was produced at a lower concentration than with HRT 3 days. However, the bioreactor was less prone to flush out. The dilution rate of 1/6 d^‐1^ gave the mean cell residence time of 6 days, as under batch conditions the yeast would be in the stationary phase at this point, this presumably contributed to the stability of the culture in the bioreactor.

**Figure 10:**
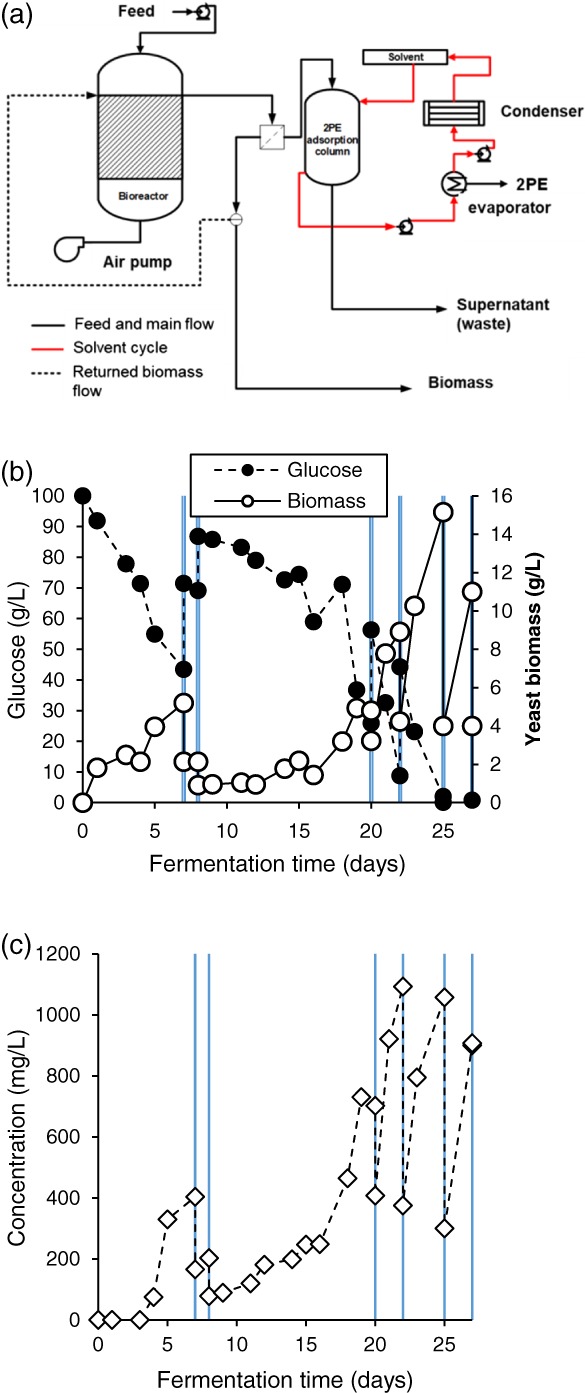
Semi**‐**continuous ex novo M. pulcherrima fermentation: (a) system set‐up; (b) glucose consumption and biomass loading; and (c) 2PE concentration. The culture was run with a dilution ratio of ½ d^‐1^ (HRT 2 days) after 20 days.

As the *ex novo* conversion of L‐phenylalanine has been demonstrated to be so efficient under optimal conditions, and high concentrations of 2PE are inhibitive towards *M. pulcherrima*, to scale this process up the fermentation was attempted in continuous mode with an external source of GAC, to prevent flush out of the carbon (Fig. 10(a)).

The input feed was fed directly into the bioreactor and the output was flowed through the adsorption column with GAC2040 (20–40 mesh) as the adsorbent. The 2PE was removed by the adsorbent and the rest of the stream sent to waste. When the adsorption in the column was at its limit, methanol was used as the solvent to leach 2PE from the column. A cross‐flow membrane (MiniKros Sampler Filter Module, Spectrum Labs Ltd) with a pore size of 750 kD and ID 1.0 mm was introduced to the overflow output to separate the biomass from the output stream allowing the aqueous phase, with 2PE, to pass through in the permeate and the yeast‐rich retentate was returned to the bioreactor to maintain the biomass. The biomass in the bioreactor was controlled by direct removal to maintain a steady concentration.

Interestingly, irrespective of sterilisation of the feed, the continuous culture became contaminated by day 5. This is presumably due to the external flow through the adsorption column being exposed to non‐sterile conditions. It seems probable that while *M. pulcherrima* can produce antibacterial compounds these were adsorbed on the activated carbon throughout the experiment. Indeed, 2PE is one of these control agents and its continual removal will risk further contamination. Another mechanism through which *M. pulcherrima* controls contamination is iron depletion by producing an iron‐binding agent, pulcherriminic acid. This prevents other microorganisms from accessing the iron. It is possible that the iron impurities in activated carbon can impair the iron depletion biocontrol mechanisms as well. The loss of antimicrobial activity removed one of the major advantages of using *M. pulcherrima* in this process.

To address this issue the culture was then run in semi‐continuous mode, in an attempt to retain high enough thresholds of the antimicrobial compounds in the bioreactor, while still retaining a non‐toxic concentration of 2PE (Fig. 10(b)).

*Metschnikowia pulcherrima* was cultured in SGJ media with L‐phe (1 g L^−1^) in the 2 L bioreactor. The fermentation was continued until the yeast entered the stationary phase at day 7. 2PE was observed from day 4. Both the lag time and the production of 2PE were slower than in the shakeflasks possibly due to the lack of sterility in the feed. At day 7, the semi‐continuous process was started with a dilution ratio of 0.5. On removal of 1 L of culture, fresh SGJ media with 2 g L^−1^ L‐phenylalanine was refilled into the fermenter and the fermentation continued.

The culture was replaced with new media on day 8 as the growth rate from previous experiments demonstrated this would be a stable system. However, the culture remained stagnant and only sluggish production of 2PE was observed. By day 20 the yeast had recovered and the culture was replaced with the new medium by dilution 0.5 on day 20. 2PE was produced in very high titres after this point with the system producing 1000 mg L^−1^ after 2–3 days, for the remaining 8 days the production of 2PE was produced steadily in this semi‐continuous fashion (Fig. 10(c)). On stabilisation of the reactor, *M. pulcherrima* was demonstrated to synthesise 2PE for approximately 900 mg L^−1^ from L‐phenylalanine 2 g L^−1^ which suggested complete conversion using this dilution ratio.

A number of other species have been investigated for the production of 2‐phenylethanol including *Pichia fermentans*, *Saccharomyces vini*, *Hansenula anomala*, *Saccharomyces cerevisiae* and *Kluyveromyces marxianus*.^1^

While *de novo* synthesis would arguably be the most economic route to 2PE production, yields are generally low, in the 100 mg L^−1^ range. The highest reported to date was from a metabolically engineered strain of *K. marxianus*, which reached 1.3 g L^−1^ when genes in the Ehrich pathway were overexpressed.^33^ The majority of studies demonstrate the productivity in the *ex novo* bioconversion of L‐phenylalanine. In particular, strains of *K. marxianus* are highly efficient producers in comparison with other species, with some reported to produce up to 0.89 g L^−1^ h^‐1^ in 41 h from molasses when 7 g L^−1^ L‐phenylananine was added.^9^ The yield was increased 3.4 times when *in situ* product removal was applied, thus avoiding growth inhibition due to 2PE accumulation in the media. Interestingly, the same study reported that a strain of *C. lusitaniae*, closely related to *M. pulcherrima*, produced up to 2.67 g L^−1^ 2PE when continual removal was used. An even higher concentration of 5.6 g L^−1^ 2PE from 9 g L^−1^ L‐phenylalanine was reported using the same *K. marxianus* strain (CBS600) when the culture conditions were optimised through simulation modeling.^34^

A wide literature is also available on 2PE production from *S. cerevisiae*. Stark *et al*. reported a *S. cerevisiae* strain to produce 2.35 g L^−1^ of 2PE from 6 g L^−1^ l‐phenylalanine in a batch culture, this was increased to 12.6 g L^−1^ in a fed‐batch culture when removal of the 2PE was applied, bringing the average 2PE production rate to 0.26 g L^−1^ h^‐1^, with a maximum value of 0.47 g L^−1^ h^‐1^.^13^ Using a hybrid system involving an immersed hollow fibre membrane module in an air‐lift reactor, the 2PE production in *S. cerevisiae* was reported to reach 18.6 g L^−1^ after 72 h in a fed‐batch culture supplemented with 9 g L^−1^ l‐phenylalanine. In this case, a decreased feeding rate and an increased aeration were key to prevent growth inhibition due to excessive ethanol production.^35^ Wang *et al*. reported an even higher productivity of 0.90 g L^−1^ h^−1^ for 65 h with *S.cerevisiae* sp. strain R‐UV3 by using continuous removal of the 2PE on a resin. This equated to a production of 0.8 mol 2PE per mol L‐phenylalanine.^36^

In comparison *M. pulcherrima* produced 1.5 g L^−1^ of 2PE from the *de novo* synthesis and in the batch can transform phenylalanine at close to the theoretical maximum. When coupled with semi‐continuous extraction, *M. pulcherrima* produced a titre of up to 14 g L^−1^ through *ex novo* conversion, or 0.61 mol moL^−1^ conversion of phenylalanine under non‐sterile conditions. This demonstrates that not only is *M. pulcherrima* a highly suitable organism for biotechnology, but is capable of producing 2PE through the *de novo* pathway in high titres, it is also able of converting phenylalanine as efficiently as other species examined to date.

## CONCLUSIONS

*Metschnikowia pulcherrima* has recently been reported as a highly suitable organism for biotechnology, as it can be grown in non‐sterile conditions and produce an array of useful products from multiple sugar sources. In this study it was demonstrated that the yeast could produce 2‐phenylethanol, the high value aroma compound used in fragrances, in high concentration from glucose and fructose. It was found that the best conditions for 2PE production were at pH 4, 20 °C with high sugar loading (100 g L^−1^). Nitrogen to carbon ratio was also found to be an important parameter for 2PE production and 1:500 was identified as being the optimal value in optimised conditions.

The 2PE production from *M. pulcherrima* was successfully scaled up to 2 L in controlled bioreactors, where the effect of aeration was examined. The yeast performed well with over 700 mg L^−1^ of 2PE and 14 g L^−1^ of yeast biomass achieved under high aeration conditions in batch mode.

*Metschnikowia pulcherrima* was also demonstrated to be an excellent 2PE producer from the *ex novo* conversion of phenylalanine. *Metschnikowia pulcherrima* was able to produce high titres of the 2PE as long as 2PE concentration in the broth was kept under the inhibition threshold (approximately 1.5–1.7 g L^−1^) for this yeast. To achieve this, *in situ* extraction was necessary. Activated carbon was demonstrated to be an excellent 2PE adsorbent, with additional PAC up to 14 g L^−1^ could be produced.

Both processes were successfully scaled to 2 L with the *de novo* production run continuously at the dilution rate of 1/3 day^‐1^ with levels over 1500 mg L^−1^ achieved before it possibly became toxic to the culture. At a lower dilution rate of 1/6 day^‐1^, the culture was more stable and no flush‐out was observed. From this steady culture over 600 mg L^−1^ of 2PE could be maintained.

The continuous fermentation of the ex‐novo process was demonstrated with *ex situ* activated carbon adsorption including 2PE leaching in the process but it was found to be susceptible to contamination, possibly due to the microbial control agents being adsorbed onto activated carbon which led to the vulnerability of the culture. However, a semi‐continuous approach was adopted in which half the working volume was replaced when the yeast reached stationary phase. Despite operating under non‐sterile conditions, no bacteria were observed and on stabilisation the bioreactor produced 0.45 g 2PE per g phenylalanine, a molar conversion of 61%.

This work demonstrates that *M. pulcherrima* is capable of producing high titres of 2PE from simple sugars or phenylalanine, and can be run successfully in a continuous mode in stirred tank bioreactors. *Metschnikowia pulcherrima* is a highly suitable platform for industrial biotechnology, and coupled with *de novo* production that is higher than any other yeast routes reported to date, could pave the way to an inexpensive microbial route to 2PE.

## Supporting information

Appendix S1. Table 1 Media formulations used in this study
**Figure S1** a) batch 2 L bioreactor set‐up for culturing M. pulcherrima and b) continuous bioreactor for 2PE production from M. pulcherrima. The condition was automatically controlled at 20 °C by cooling coil and heating blanket and pH 4 by the automatic addition of HCl 1 M and NaOH 1 M
**Figure S2** Dissolved oxygen profiles for culturing M. pulcherrima in 2 L bioreactors at 20 °C, pH 4 under batch conditions.
**Figure S3** Synergistic toxicity of 2‐phenylethanol and ethanol to M. pulcherrima in 96‐well plates at 20 °C at pH 4. (n = 6) (red = best growth, blue = worst growth)
**Figure S4** Effect of dodecane (DDC) and oleyl alcohol (OA) on yeast biomass
**Figure S5** Temperature, pH and dissolved oxygen in the de novo batch production
**Figure S6** Addition of granulated activated carbon to the ex‐novo batch production of 2PE.
