# Supplementary material for: Elevated production of the aromatic fragrance molecule, 2‐phenylethanol, using Metschnikowia pulcherrima through both de novo and ex novo conversion in batch and continuous modes
Source: J Chem Technol Biotechnol. 2018 Mar 25;93(8):2118–30. doi: 10.1002/jctb.5597 (PMC6055805; doi:10.1002/jctb.5597)
Supplement: Supplementary file 1 — Appendix S1. Table 1 Media formulations used in this study Figure S1 a) batch 2 L bioreactor set‐up for culturing M. pulcherrima and b) continuous bioreactor for 2PE production from M. pulcherrima. The condition was automatically controlled at 20 °C by cooling coil and heating blanket and pH 4 by the automatic addition of HCl 1 M and NaOH 1 M Figure S2 Dissolved oxygen profiles for culturing M. pulcherrima in 2 L bioreactors at 20 °C, pH 4 under batch conditions. Figure S3 Synergistic toxicity of 2‐phenylethanol and ethanol to M. pulcherrima in 96‐well plates at 20 °C at pH 4. (n = 6) (red = best growth, blue = worst growth) Figure S4 Effect of dodecane (DDC) and oleyl alcohol (OA) on yeast biomass Figure S5 Temperature, pH and dissolved oxygen in the de novo batch production Figure S6 Addition of granulated activated carbon to the ex‐novo batch production of 2PE. [file JCTB-93-2118-s001.doc]

**Production of the fragrance molecule, 2-phenylethanol, from the *de-novo* conversion of glucose using *Metschnikowia pulcherrima* in both batch and continuous modes**

Supporting information

Tanakorn Chantasuban, Fabio Santomauro and Christopher Chuck*

Table 1 Media formulations used in this study

| **Formula** | **SGJ Media (g/L)** | **SGGJ**  **Media**  **(g/L)** |
| --- | --- | --- |
| **glucose (+)** | 70 | 100 |
| **fructose** | 30 |  |
| **tartaric acid** | 7 | 7 |
| **malic acid** | 10 | 10 |
| **(NH_4_)_2_HPO_4_** | 0.67 | 0.67 |
| **KH_2_PO_4_** | 0.67 | 0.67 |
| **MgSO_4_ *7H_2_O** | 1.5 | 1.5 |
| **NaCl** | 0.15 | 0.15 |
| **FeSO_4_ * 7H_2_O** | 0.021 | 0.021 |
| **ZnSO_4_ *7H_2_O** | 0.0075 | 0.0075 |
| **CaCl_2_** | 0.15 | 0.15 |


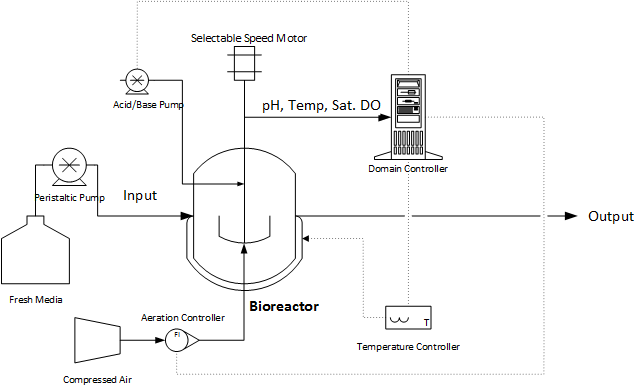


**Figure S1** a) batch 2L bioreactor set-up for culturing *M. pulcherrima* and b) continuous bioreactor for 2PE production from *M. pulcherrima*. The condition was automatically controlled at 20°C by cooling coil and heating blanket and pH4 by the automatic addition of HCl 1M and NaOH 1M


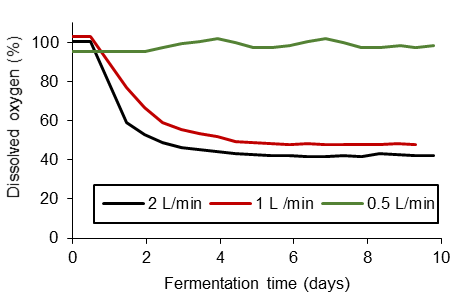


**Figure S2** Dissolved oxygen profiles for culturing *M. pulcherrima* in 2L bioreactors at 20 °C, pH4 under batch conditions.


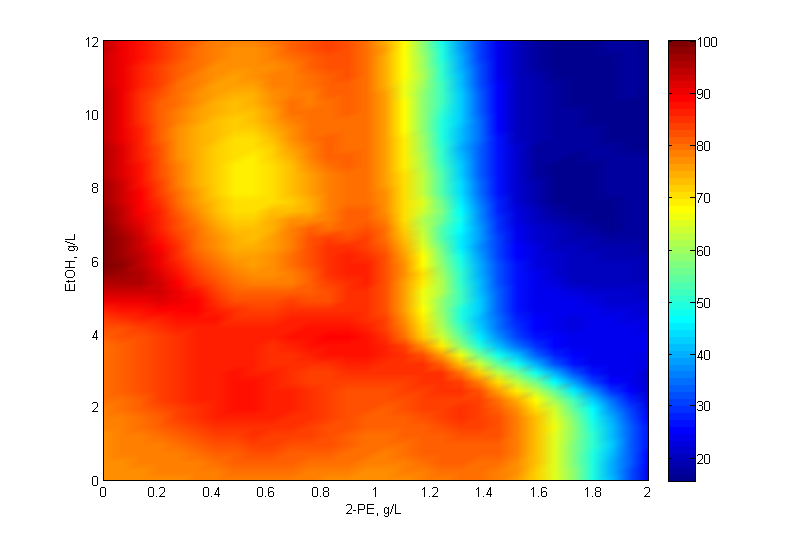


**Figure S3** Synergistic toxicity of 2-phenylethanol and ethanol to M. pulcherrima in 96-well plates at 20 °C at pH 4. (n=6) (red = best growth, blue = worst growth)


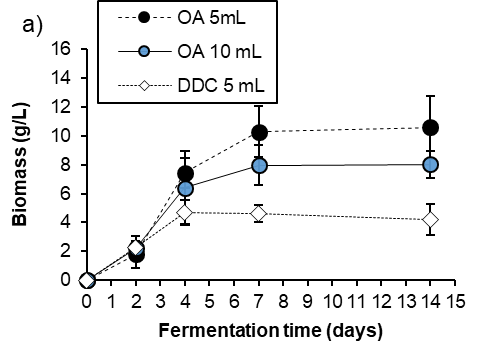


**Figure S4** Effect of dodecane (DDC) and oleyl alcohol (OA) on yeast biomass


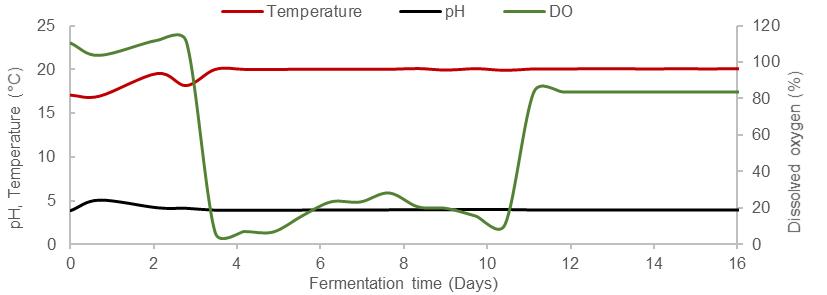


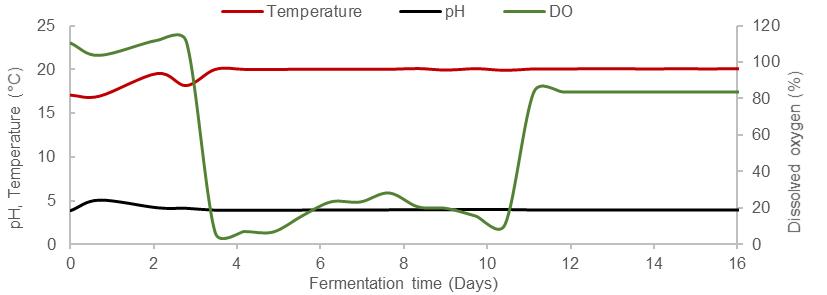


**Figure S5** Temperature, pH and dissolved oxygen in the de novo batch production


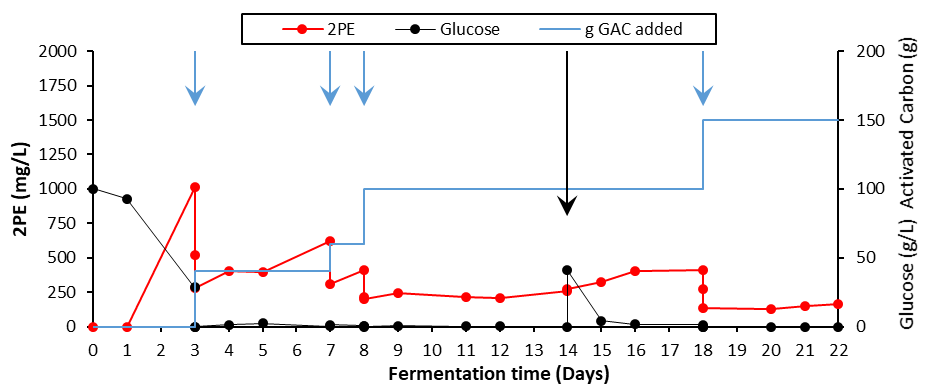


**Figure S6** Addition of granulated activated carbon to the ex-novo batch production of 2PE.
